# Endometrial preparation for frozen-thawed embryo transfer in an artificial cycle: transdermal versus vaginal estrogen

**DOI:** 10.1038/s41598-020-57730-3

**Published:** 2020-01-22

**Authors:** Romain Corroenne, Hady El Hachem, Caroline Verhaeghe, Guillaume Legendre, Cecile Dreux, Pauline Jeanneteau, Philippe Descamps, Pascale May-Panloup, Pierre-Emmanuel Bouet

**Affiliations:** 1 Department of Reproductive Medicine, Angers University Hospital, 4 rue Larrey, 49100 Angers, France; 2 Department of Reproductive Medicine, Clemenceau Medical Center, Clemenceau Street, Beirut, Lebanon

**Keywords:** Endocrinology, Outcomes research

## Abstract

The objective was to compare the endometrial thickness (ET) in a frozen embryo transfer (FET) cycle between transdermal and vaginal estrogen. Our secondary objectives were to compare the patient satisfaction and the pregnancy outcomes. Prospective monocentric cohort study between 01/2017 and 12/2017 at a single institution. Choice of administration was left to the patient. 119 cycles had transdermal estrogen (T-group) and 199 had vaginal estrogen (V-group). The ET at 10 ± 1 days of treatment was significantly higher in the T-group compared to the V-group (9.9 vs 9.3 mm, p = 0.03). In the T-group, the mean duration of treatment was shorter (13.6 vs 15.5 days, p < 0.001). The rate of cycle cancelation was comparable between the two groups (12.6% vs 8.5%, p = 0.24). Serum estradiol levels were significantly lower (268 vs 1332 pg/ml, p < 0.001), and serum LH levels were significantly higher (12.1 ± 16.5 vs 5 ± 7.5 mIU/ml, p < 0.001) in the T-group. Patient satisfaction was higher in the T-group (p = 0.04) and 85.7% (36/42) of women who had received both treatments preferred the transdermal over the vaginal route. Live birth rates were comparable between the two groups (18% vs 19%, p = 0.1). Transdermal estrogen in artificial FET cycles was associated with higher ET, shorter treatment duration and better tolerance.

## Introduction

Frozen embryo transfers (FET) constitute nowadays an integral part of an *in vitro* fertilization (IVF) program, and their use has gradually increased over the past decades. The latest annual report of the European national registries indicates there were 192 017 FET cycles in 2014, a 24% increase compared to 2013, while in France, approximately 32 000 FET cycles were performed in 2016, which constitutes a 140% rise compared to 2012. This increase is the consequence of the improvement of cryopreservation techniques and the subsequent results, as well as the reassuring long term safety data^1^. Indeed, the latest data from the Centers for Disease Control (CDC) show that across the USA, where FET account for more than 32% of all assisted reproductive technologies, pregnancy and live birth rates following FET are comparable and sometimes better than fresh cycles^1^. Moreover, the indications for FET have increased, mainly due to more single embryo transfers (SET) being performed worldwide (63.6% in 2015 versus 53.2% in 2012 in France^1–3^), more agonist triggering for risk of ovarian hyperstimulation syndrome, more freeze-all strategies, and increased use of preimplantation genetic testing (PGT)^1,4^.

A FET can be performed in a natural cycle, a modified natural cycle (with ovulation triggering), an artificial cycle using treatment with exogenous estrogen and progesterone, and a stimulated cycle using exogenous gonadotrophins^5–7^. Each of these methods has its advantages and drawbacks. Natural cycles allow the patients to have a treatment-free transfer, but could be problematic in terms of scheduling the activity in an IVF unit since the transfer date is dictated by the patient’s ovulation, and cannot be offered to women with irregular cycles. These two problems can be resolved with the use of an artificial cycle, the most commonly used FET protocol worldwide. Stimulated cycles are associated with a higher cost, and more treatment related side effects, and are usually offered in second line and in specific cases. Despite the many differences, overall pregnancy and live birth rates seem to comparable between all these protocols^8,9^.

Several preparation methods have been proposed for FET in artificial cycles. Exogenous estrogen is administered early in the follicular phase in order to induce endometrial proliferation and inhibit spontaneous ovulation, with progesterone added days before the embryo transfer^9–12^. Estrogen can be given as an oral or a vaginal tablet, a transdermal patch, and a subcutaneous or intramuscular injection, with no significant differences in outcomes^13–15^. A 2014 international survey that included 161 fertility specialists from 35 countries showed that 86% of participants used the oral route, followed by the transdermal (8%), vaginal (3%), intramuscular (2%) and other routes (1%)^15^. Compared to the oral route, the transdermal and vaginal route offer several advantages: A higher bioavailability since it bypasses the intestinal and hepatic metabolism, thus decreasing the risk of conversion of estradiol to estrone, and a more stable plasma concentration^11,12,14,16,17^. Several studies have compared the oral route to the vaginal or transdermal, but to date, no study has compared the outcomes and side effects between the transdermal and vaginal routes.

The main objective of our study was to determine if there is any difference in endometrial thickness in a FET cycle between transdermal and vaginal estrogen. Our secondary objectives were to compare the global patient satisfaction and the undesirable side effects between the two protocols, as well as the pregnancy outcomes and the cancelation rates.

## Results

**Figure 1:**
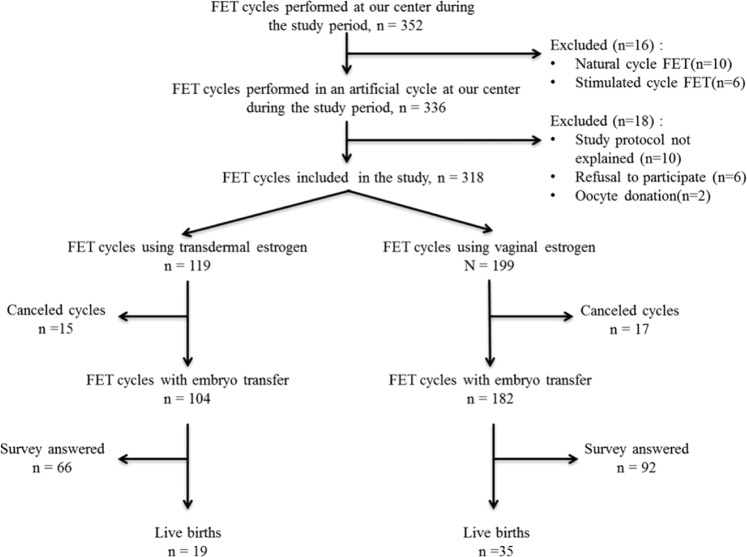
Flow chart of the study.

During the course of the study, we performed 352 FET cycles: 336 in an artificial cycle, 10 in a natural cycle and 6 in a stimulated cycle. Out of the 336 artificial cycles, 18 were excluded: 10 were missed by the treating physician or nurse, 6 refused to participate and 2 were from egg donation cycles. Overall, we included 318 cycles in 215 patients, 119 (37.4%) using transdermal estrogen (T group), and 199 (62.6%) using vaginal estrogen (V group). 87.4% of cycles (104/119) led to an embryo transfer in the T group and 91.4% (182/199) in the V group. The questionnaire was completed in 63% of cases in the T group (66/104) and 50.5% (92/182) in the V group. None of the patients was lost to follow-up and data concerning pregnancy outcomes were available for all included cycles. The flow chart is shown in Fig. 1.

Patients’ characteristics are shown in Table 1. The two groups were comparable for age, body mass index (BMI), basal FSH, type and cause of infertility, and smoking status. One patient in the T group and 2 patients in the V group had a previous history of a uterine septum that was hysteroscopically resected, and there were no other müllerian anomalies in both groups. The characteristics of the embryos transferred were also comparable between the two groups (single or double ET, top quality embryos, day of transfer) (Table 2). Cycles’ characteristics are shown in Table 3.

**Table 1:** Patients’ characteristics.

	Transdermal group N = 82	Vaginal group N = 133	p
Age (years)	33 ± 4.27	33 ± 4.89	0.24
Body Mass Index (kg/m^2^)	24.5 ± 5.742	24.2 ± 5.053	0.89
Tobacco use	17 (20.7)	35 (26.3)	0.32
Primary infertility	58 (70.7)	98 (73.7)	0.64
Origin of infertility			0.85
• Female factor	29 (35.4)	41 (30.8)	
• Male factor	21 (25.6)	34 (25.6)	
• Mixed	15 (18.3)	24 (18)	
• Unexplained	17 (20.7)	34 (25.6)	
FSH basal (mIU/mL)	6.7 ± 2.3	6.5 ± 2.2	0.65

Data are expressed as n (%) or mean ± standard deviation.

**Table 2:** Embryo transfer characteristics.

	Transdermal group N = 104	Vaginal group N = 182	p
Difficult embryo transfer	1 (1)	2 (1.1)	0.91
Single embryo transfer	74 (71.1)	126 (69.2)	0.73
Double embryo transfer	30 (28.8)	56 (30.8)	0.73
“Top” quality embryo transfer	23 (22.1)	52 (28.6)	0.23
Loss of embryo viability during thawing	2 (1.9)	3 (1.6)	1
Day 2 embryo transfer	47 (45.2)	85 (46.7)	0.80
Day 3 embryo transfer	45 (43.3)	78 (42.9)	0.95
Day 5 embryo transfer	10 (9.6)	18 (9.9)	0.94
Day 6 embryo transfer	2 (1.9)	1 (0.5)	0.30

Data are expressed as n (%).

**Table 3:** FET cycle characteristics.

	Transdermal group N = 119	Vaginal group N = 199	p
Endometrial thickness on day 10 ± 1 of treatment (mm)	9.9 ± 2.1	9.3 ± 2.3	0.03
Ultrasound reassessment for endometrial thickness < 8 mm at day 10 ± 1 of treatment	12 (10.1)	61 (30.6)	< 0.001
FET performed with endometrial thickness < 8 mm	3 (2.5)	16 (8)	0.04
Mean duration of treatment (days)	13.6 ± 2.7	15.5 ± 3.2	< 0.001
Estradiol level on day of first ultrasound (pg/mL)	268 ± 454	1332 ± 769	< 0.001
LH level on day of first ultrasound (mIU/mL)	12.1 ± 16.5	5 ± 7.5	< 0.001
Progesterone level on day of first ultrasound (ng/mL)	0.6 ± 1.3	0.5 ± 1.4	0,61
Canceled cycles	15 (12.6)	17 (8.5)	0.24
• Spontaneous ovulation	9 (7.6)	4 (2)	0.02
• Insufficient endometrial thickness	1 (0.8)^b^	3 (1.5)^c^	1
• Loss of embryo viability during thawing	2 (1.7)	3 (1.5)	1
• Other ^a^	3 (2.5)	7 (3.5)	0.62

Data are expressed as n (%) or mean ± standard deviation.

^a^These cancelations were due to fever occurring during treatment, deep pelvic pain secondary to endometriosis, or a history of recent travel to a region with risk of Zika virus endemic area.

^b^Idiopathic thin endometrium.

^c^No etiology was found for the thin endometrium in two of these patients. One patient had a positive history of synechia that occurred following a septum resection, and was hysteroscopically treated.

### Endometrial thickness

The endometrial thickness on day 10 ± 1 was significantly higher in the T group compared to the V group (9.9 mm vs 9.3 mm, p = 0.03) and significantly fewer patients in the T group required a second ultrasound reassessment for endometrial thickness (10.1% vs 30.6%, p < 0.001). All patients who had a transfer had an endometrial thickness ≥ 8 mm, and no patient had a transfer with a thickness between 6 and 7 mm.

### Duration of treatment

The duration of treatment was significantly shorter in the T group compared to the V group (13.6 ± 2.7 vs 15.5 ± 3.2 days, p < 0.001), and the rate of cycles when the ET was performed despite an endometrial thickness < 8 mm was significantly lower in the T group compared to the V group (2.5% vs 8%, p = 0.04, respectively).

### Plasma hormonal level

Serum estradiol levels on the day of the first ultrasound were significantly lower in the T group (268 ± 454 vs 1332 ± 769 pg/ml, p < 0.001), while serum LH levels were significantly higher (12.1 ± 16.5 vs 5 ± 7.5 mIU/ml, p < 0.001) compared to the V group. Progesterone levels were comparable between the T and the V group (0.6 ± 1.3 vs 0.5 ± 1.4, p = 0.61, respectively).

### Cycle cancelation

The rate of cycle cancelation was comparable between the T and the V group (12.6% vs 8.5%, p = 0.24) (Table 3). However, when looking at the cause of cycle cancelation, there was significantly more spontaneous ovulation in the T group (7.6% (9/119) vs 2% (4/199), p = 0.02), but the other causes were comparable between the two groups (Table 3).

### Outcomes

Cycle outcomes are listed in Table 4. There were no significant differences in the CPR per cycle started [26.9% (32/119) vs 24.6% (49/199), p = 0.65] and per ET [30.8% (32/104) vs 26.9% (49/182), p = 0.25], in the biochemical pregnancy loss rate [9.4% (3/32) vs 6.1% (3/49), p = 0.58], in the clinical pregnancy loss [31.2% (10/32) vs 22.4% (11/49), p = 0.38] and the LBR, per cycle started [16% (19/119) vs 17.6% (35/199), p = 0.71] and per ET [18.3% (19/104) vs 19.2% (35/182), p = 0.84] between the transdermal and vaginal group, respectively.

**Table 4:** Cycles outcomes according to type of estrogen used.

	Transdermal group	Vaginal group	p
Clinical pregnancy rate			
• Per started cycle	26.9 (32/119)	24.6 (49/199)	0.65
• Per embryo transfer	30.8 (32/104)	26.9 (49/182)	0.25
Adverse pregnancy outcomes			
• Biochemical pregnancy loss*	9.4 (3/32)	6.1 (3/49)	0.58
• Clinical pregnancy loss	31.2(10/32)	22.4 (11/49)	0.38
Live birth rate			
• Per started cycle	16 (19/119)	17.6 (35/199)	0.71
• Per embryo transfer	18.3 (19/104)	19.2 (35/182)	0.84

Data are expressed as percentage (n/total).

*biochemical pregnancy loss was defined after a first positive β-human Chorionic Gonadotropin (β-hCG) test (>25 mIU/mL), following by decreasing β-hCG levels without clinical pregnancy.

### Satisfaction

The overall satisfaction score was significantly higher in the T group compared to the V group (8.2 vs 7.4, p = 0.04) (Table 5). Moreover, significantly more patients reported not having any undesirable side effect [39.4% (26/66) vs 23.9% (22/92), p = 0.04], and fewer patients reported at least one or many drawbacks [24.2% (16/66) vs 41.3% (38/92), p = 0.03] in the T group compared to the V group. The two most common side effects in the V group were breast pain (45.7%) and fatigue (43.5%), and both were significantly lower in the T group (9.1%, p < 0.001 and 27.3%, p = 0.037, respectively). Irritability was the third most common side effect in the V group (26.1%), with no difference observed in the T group (15.2%, p = 0.1). On the other hand, there was significantly more redness/itching at the application site in the T group compared to the V group (16.7% vs 1.1%, p < 0.001).

**Table 5:** Outcomes of patient satisfaction survey.

	Transdermal group N = 66	Vaginal group N = 92	p
Global satisfaction score (/10)	8.2 ± 2	7.4 ± 2.2	0.04
No undesirable side effect noted	26 (39.4)	22 (23.9)	0.04
One or many drawbacks of treatment noted	16 (24.2)	38 (41.3)	0.03
Undesirable side effects noted by patients			
• Irritability	10 (15.2)	24 (26.1)	0.1
• Headaches	9 (13.6)	12 (13.0)	0.91
• Insomnia	6 (9.1)	8 (8.7)	0.93
• Nausea	6 (9.1)	13 (14.1)	0.34
• Diarrhea	2 (3.0)	4 (4.3)	0.67
• Redness, itching at the application site	11 (16.7)	1 (1.1)	<0.001
• Acne	4 (6.1)	14 (15.2)	0.07
• Dry skin	5 (7.6)	11 (12.0)	0.37
• Back pain	4 (6.1)	8 (8.7)	0.54
• Mastalgia	6 (9.1)	42 (45.7)	<0.001
• Fatigue	18 (27.3)	40 (43.5)	0.037
• Other	8(12.1)	10(10.9)	0.81
Mean number of side effects noted	1.3 ± 1.5	2.5 ± 2.3	<0.001

Data are expressed as n (%) or mean ± standard deviation.

Finally, 64.6% of patients (42/65) in the T group had already used vaginal estrogen for FET in previous cycles. Of those, 85.7% (36/42) stated that they preferred the transdermal route over the vaginal route; 12% (5/42) declared they had more side effects with the transdermal route; 33% (14/42) had more side effects with the vaginal route; 17% (7/42) had the same rate of side effects with the two routes; and 38% (16/42) declared having no side effects with any of the two.

## Discussion

Our study has showed that transdermal estrogen was associated with higher endometrial thickness, shorter treatment duration, fewer side effects and higher patient satisfaction, but a higher cycle cancelation rate, compared to the vaginal route for endometrial preparation in artificial FET cycles. No differences were observed in clinical pregnancy, clinical pregnancy loss, biochemical pregnancy loss and live birth rate between the two routes.

Endometrial thickness is a critical element for the success of an IVF cycle, in fresh and frozen transfers. Several studies have assessed its impact on cycle outcomes, and the threshold used to consider an endometrial thickness as acceptable varies between 7, 8 or 9 mm according to studies. El-Toukhy *et al*., in one of the earliest studies on endometrial thickness in FET, reported significantly higher LBR in cycles with an endometrial thickness between 9 and 14 mm compared to 7–8 mm (25% vs 14%, p = 0.002)^18^. In a retrospective analysis of 2997 FET cycles over a 3 year period, Bu Z *et al*. showed that an endometrial thickness on transfer day between 9 and 14 mm was associated with significantly better LBR compared to ≤ 8 mm after adjusting for age, BMI, baseline FSH, FET protocol and number of embryos transferred (aOR = 1.5; 95% CI :1.16–1.95)^19^. The largest report to date, a retrospective cohort study of the Canadian ART registry^20^ included 18 942 FET cycles, and found that CPR and LBR declined with each mm decrease below 7 mm. However, no comparisons were made between thicknesses ≥ 8 mm (85.9% of cycles).

In our study, mean endometrial thickness was > 9 mm in both groups, which is in accordance with the acceptable thickness for transfer in the literature, but it was significantly higher in the T group (9.9 vs 9.3 mm, p = 0.03). Moreover, there were significantly fewer cycles where the transfer was performed despite an endometrial thickness < 8 mm in the T group. Even though the increase in thickness might have been inconsequential in this study, it could be hypothetically useful in cases with a thin endometrium ( < 7 mm), in order to reach the acceptable cut-off.

On the other hand, concerning endometrial thickness, our study showed that the transdermal route is significantly more efficient than the vaginal route for achieving the required threshold. We did not find any other study in the literature comparing these two routes, but found several studies comparing the transdermal to the oral route, with contradictory results. One randomized controlled trial (RCT) found that endometrial thickness was significantly higher with transdermal compared to oral treatment^21^, while two other RCTs failed to find any significant difference in endometrial thickness between the two routes^22,23^.

In all, patients who received transdermal estrogen had a significantly shorter duration of treatment (13.6 ± 2.7 vs 15.5 ± 3.2 days, p < 0.001), with fewer cases requiring ultrasound reassessment (10.1% vs 30.6%, p < 0.001), compared to the vaginal route. By offering a shorter duration of treatment and fewer clinic visits, the transdermal route might be more attractive for patients than the vaginal route.

Mean plasma estradiol levels were more than five times higher in the vaginal group compared to the transdermal group (268 vs 1332 pg/ml). This difference was expected and can be explained by the different metabolism of these two routes: Both bypass the first pass hepatic metabolism, and vaginal results in very high serum levels because of an important absorption through the vaginal mucosa while transdermal produces the most stable steady-state levels^12,24^. High estradiol levels are associated with many drawbacks: a higher vascular risk^25,26^, lower LBR in FET, and higher risk of small for gestational age, abnormal placentation and preeclampsia in the ensuing pregnancy after fresh embryo transfers^27–29^. Moreover, the more physiologic hormonal milieu found in FET cycles is one of the main arguments in favor of frozen over fresh embryo transfers. Therefore, the lower plasma estradiol levels with transdermal estrogen could be an added advantage over the vaginal route.

It seems contradictory to have a significantly lower serum estradiol levels in the T group with a significantly shorter duration of treatment. One possible explanation is that very high levels of serum estradiol, as those obtained with the vaginal route, could have an antiproliferative effect. Indeed, the estradiol nuclear receptors $\beta (ER\beta )$, as well as the G protein coupled transmembrane receptors located in the endoplasmic reticulum, have been shown to activate cellular proliferation with low serum estradiol levels, while they could have an antiproliferative effect with high serum estradiol levels^30–32^.

There were more cycle cancelations because of spontaneous ovulation in the T group compared to the V group (7.6% vs 2%, p = 0.02). This can be explained by the lower serum estradiol levels with transdermal treatment, leading to less efficient inhibition of the hypothalamic-pituitary axis (HPO). Indeed, the serum LH levels were significantly higher on the day of ultrasound assessment in the T group (12.1 vs 5 mIU/mL, p < 0.001). This drawback of transdermal estrogen could be overcome with pre-treatment pituitary down-regulation using a Gonadotropin releasing hormone agonist (GnRHa). Indeed, down-regulation with GnRHa is commonly used in artificial FET cycles, no matter the type of estrogen used and without any negative impact on endometrial thickness^18,33^. However, results have not shown any added benefits when using oral or vaginal estradiol, where the rates of spontaneous ovulation are already low^34^. However, it could be beneficial when using transdermal estrogen for FET since spontaneous ovulation seems to be higher. Another option to reduce the spontaneous ovulation rate with transdermal treatment would be to start treatment on day 2–3 with two patches of 100 µg instead of one, thus increasing the serum estradiol levels and the negative feedback on the HPO axis.

We did not find any difference in CPR, clinical pregnancy loss, biochemical pregnancy loss and LBR between the transdermal and vaginal groups. The CPR and LBR obtained in our study are in accordance with the French national and the European rates following FET^35,36^. Several studies have already shown that the type of estrogen treatment does not influence the pregnancy outcomes in FET^21,34,37^, but our study is the first to directly compare the transdermal and vaginal route, and confirm the lack of difference between the two.

Our study has shown a better tolerance of transdermal estrogen compared to vaginal estrogen. Indeed, the global satisfaction score was significantly higher and the rate of undesirable side effects was significantly lower in the T group. Moreover, 85.7% of the patients who had received the two treatments in two different cycles stated a preference for the transdermal route. The higher rate of undesirable side effects could be explained by the higher serum estradiol levels in the vaginal group. On the other hand, the only side effect that was significantly higher with transdermal treatment was redness/itching at the application site, a commonly cited side effect of patches^21,22^. Patient tolerance is an essential factor to be taken into consideration when choosing a treatment for FET, considering patients have to be highly compliant for a period of 8 weeks when pregnant. Only one study in the literature compared patients’ tolerance and compliance between the transdermal and oral route, and found that oral treatment was more comfortable for patients with fewer side effects^21^.

To the best of our knowledge, our study is the first to compare endometrial thickness and cycles outcomes, as well as patient satisfaction and tolerability, between transdermal and vaginal estradiol for endometrial preparation in FET cycles. Moreover, our clinical findings were corroborated by biologic data, further validating our conclusions. Our main limitations were the lack of randomization and the monocentric design. Furthermore, our study included day 2–3 (90%) and day 5–6 (10%) embryo transfers, and was underpowered to compare pregnancy and live birth rates. Patient’s satisfaction survey answers could have been influenced by a previous cycle outcome using one route or the other. Finally, it would have been interesting to add the oral route to the comparison, but we do not use oral estrogen for endometrial preparation in our center.

In conclusion, our study has shown that transdermal estrogen was associated with higher endometrial thickness and shorter treatment duration when compared with vaginal estrogen for endometrial preparation before FET, but higher cycle cancelation because of spontaneous ovulation. Moreover, transdermal estrogen was better tolerated by patients with significantly fewer side effects. Finally, pregnancy outcomes were comparable between the two. These findings suggest that transdermal estrogen should be offered first for endometrial preparation before FET. A pretreatment injection of GnRH agonist could be added to decrease the rate of spontaneous ovulation and cycle cancelation.

## Material and Methods

We performed a unicentric prospective cohort study at the department of reproductive medicine of the Angers university hospital, between January 1^st^ 2017 and December 31^st^ 2017. The study was registered in the clinical trial database at Clinicaltrials.gov (NCT03518528) and was approved by our institutional review board (Centre hospitalier universitaire d’Angers - No 2017–19). All patients were briefed on the study and signed an informed consent before inclusion. All methods were performed in accordance with the relevant guidelines and regulations.

The inclusion criteria were: (1) age ≥ 18 years; (2) all patients having one or many frozen embryos following IVF/ICSI; (3) all patients undergoing a FET in an artificial cycle; (4) patients who signed the informed consent. We excluded from the study all patients: (1) who refused to sign the consent form; (2) who were given a specific treatment without being given the option between the two routes; (3) who had a FET performed in natural or stimulated cycles; (4) who had a FET following oocyte donation; (5) under legal guardianship; (6) non beneficiaries of a social security scheme; (7) non-French speaking.

Before January 1^st^ 2017, all patients undergoing FET in an artificial cycle at our center received vaginal estrogen for endometrial preparation: 2 vaginal tablets of estrogen 2 mg (Provames® 2 mg, Sanofi) inserted daily, at bedtime. Estrogen was started on day 2 of the menstrual cycle and continued at the same dose until the serum human Chorionic Gonadotropin (hCG) testing, and until 8 weeks of pregnancy when positive. On January 1^st^ 2017, we implemented a new protocol and started using transdermal estrogen:1 patch of Estradiol 100 µg (Vivelledot®, Novartis Pharma SAS), on day 2 of the menstrual cycle, replaced on the seventh day of the cycle with 2 other patches, in order to mimic the rising estradiol levels observed in a natural cycle. The patches were changed every 4 days, and treatment continued until the serum hCG test and until 8 weeks of pregnancy when positive. The patients were instructed to apply the patches on a clean and dry skin, in the lower abdomen region or on the hips, based on their preference.

The two types of treatments were explained to the patients by the treating physician during the initial consultation in the period before the FET cycle, and by the departments’ nurses during the telephone consultation that is systematically performed for all patients in the month preceding the FET cycle. Having received the information, the choice between the vaginal route (V group) and the transdermal route (T group) was left to the patient, and the drug prescriptions were handed accordingly. Several factors could have influenced the decision towards the vaginal route: the habit of the prescribing physician and nurse since it is the original protocol, the patient’s preference based on a history of successful FET using the vaginal route, or their refusal of a “new” protocol recently put in place. On the other hand, the decision to use the transdermal route could have been influenced by the treating physician or nurse’s motivation to implement the new protocol, the patient’s aversion for vaginal pills or a previous history of a failed FET using the vaginal route.

All patients had the first ultrasound to check the endometrial thickness on day 10 ± 1, along with serum estradiol, LH and progesterone levels. The endometrial thickness was measured using a 2D transvaginal ultrasound probe (Voluson E8, General Electrics®, Boulogne-Billancourt, France), in the median longitudinal plane. The distance was measured between the two calipers placed at the outer lining of the endometrial–myometrial interface from the anterior to the posterior wall of the uterus. The measurements were performed by three attending physicians and two senior fellows. When the endometrial thickness was ≥ 8 mm, intravaginal progesterone was started for luteal phase support (ESTIMA Gé®, Effik) (200 mg, 1 vaginal pill three times daily), until the hCG test and until 8 weeks of pregnancy when positive. The day of transfer was programed according to the age of the embryo, the day of progesterone start, the patient’s availability, and the clinical activity in the department. Progesterone was started on the night of the theoretical day of oocyte retrieval, meaning day 3 embryos were transferred on day 4 of progesterone, and blastocysts transferred on day 6 of progesterone. When the endometrial thickness was < 8 mm on the first ultrasound, treatment was increased to 3 vaginal tablets of estrogen 2 mg (total 6 mg) or to 3 patches of Estradiol 100 µg (total 300 µg), and a follow-up ultrasound programmed 5 to 6 days later. The uterine artery Doppler was measured bilaterally for all patients, and those with a pulsatility index > 3 on at least one of the arteries received oral treatment with pentoxifylline, 400 mg daily, until the hCG test. The FET was performed whenever the endometrial thickness was ≥ 7 mm, but a thickness between 6 and 7 mm was considered acceptable for patients with a history of synechia or endometrial surgery, and patients with previous canceled FET. Cycle cancelation was defined as a cycle abandoned before the embryo transfer. Cycles were canceled when the endometrial thickness remained low (<6–7 mm) despite the treatment adjustment, when a spontaneous ovulation occurred (diagnosed by a high progesterone level with the presence of a corpus luteum on the ultrasound), or when other unexpected external factors occurred that would compromise the chances of success (infectious disease, non-observance errors, exacerbation of pelvic pain due to endometriosis, Zika virus infection risk…). Cycles could also be canceled even after progesterone supplementation was started, such as cases where the embryos did not survive the thaw procedure.

All embryo transfers were performed by the senior physicians in the department, under pelvic ultrasound guidance, with a soft catheter (Elliocath®, CCD). A rigid catheter (Set TDT®, CCD) was used for difficult transfer cases. At our center, we transfer embryos on day 2, 3 or 5, depending on the case. All embryos had been cryopreserved by vitrification, and were thawed the morning of the planned transfer and transferred on the same day they had been cryopreserved. A “top” embryo was defined as an embryo with at least 4 blastomeres on Day 2 and 8 blastomeres on Day 3, identical in size and form, with < 10% fragmentation and no multinucleation, according the BLEFCO classification (13). Blastocysts were classified according to the Gardner and Schoolcraft criteria^38^.

On the day of embryo transfer, an anonymous questionnaire was handed to all included patients, depending on the treatment they received. Patients who had vaginal estrogen were asked to: quote their satisfaction from 0 (not satisfied at all) to 10 (totally satisfied), describe if they had any undesirable side effects and what were they, and specify if there were any drawbacks related to the treatment (Supplementary material 1). Patients who received the transdermal route were asked the same questions, with extra questions reserved for patients who had already received vaginal estrogen for another FET prior to this one. These patients were asked to state their preference between the two routes, and which one had more side effects (Supplementary material 2). Each patient received only one questionnaire and was only included once if she received the same protocol twice, but twice if she received the two protocols (one for each group).

When a FET was performed, serum bHCG levels were tested 14 days after the initiation of progesterone, and repeated 48 hours later. When positive, the test was repeated seven days later. Clinical pregnancy was defined as a positive fetal heartbeat at the first ultrasound on 7 weeks gestational age (GA), a clinical miscarriage as a loss of a recognized pregnancy < 20 weeks GA, and live birth as delivery of a viable infant ≥ 25 weeks GA. A biochemical pregnancy loss was defined after a first positive bHCG ( > 25 mIU/mL), following by decreasing bhCG levels without clinical pregnancy.

Our primary outcome was endometrial thickness on day 10 ± 1 of treatment. Our secondary outcomes were the clinical pregnancy rate (CPR), live birth rate (LBR), clinical miscarriage loss, biochemical pregnancy loss, the hormonal profile (serum estradiol, progesterone and LH) on the first ultrasound, the cancelation rate, the number of days of treatment before the FET, and the satisfaction rate as measured by our survey.

Statistical analysis was performed using IBM® SPSS 22.0 (New York, USA). Continuous variables were expressed as mean values and standard deviations (SD) and compared with Student’s t-test, whereas categorical variables were expressed as percentages and compared with chi-squared or Fisher’s exact test for small samples ( < 10). The number of patients needed to treat was determined as follows: We calculated that a sample size of 90 cycles in each group (total 180) was required to detect a difference of 1 mm of endometrial thickness between the two groups, with a standard deviation of 2 mm, with a power of 90%, a 5% α risk, and a 5% drop-out. We performed an intention to treat analysis by including all started cycles and a per protocol analysis by including only cycles where the FET was performed.

## Supplementary information

Supplementary Material.

## Data Availability

The dataset generated during the current study can be made available upon request to the corresponding author.
