# Supplementary material for: Endometrial preparation for frozen-thawed embryo transfer in an artificial cycle: transdermal versus vaginal estrogen
Source: Sci Rep. 2020 Jan 22;10:985. doi: 10.1038/s41598-020-57730-3 (PMC6976623; doi:10.1038/s41598-020-57730-3)
Supplement: Supplementary file 1 — Supplementary Material. [file 41598_2020_57730_MOESM1_ESM.docx]

**Title:** Endometrial preparation for frozen-thawed embryo transfer in an artificial cycle: transdermal versus vaginal estrogen

**Authors:**

Romain Corroenne^1*^, Hady El Hachem^2^, Caroline Verhaeghe^1^, Guillaume Legendre^1^, Cecile Dreux^1^, Pauline Jeanneteau^1^, Philippe Descamps^1^, Pascale May-Panloup^1^, Pierre-Emmanuel Bouet^1^

^1^ Department of Reproductive Medicine, Angers University Hospital, 4 rue Larrey, 49100 Angers, France.

^2^ Department of Reproductive Medicine, Clemenceau Medical Center, Clemenceau Street, Beirut, Lebanon.

**Supplementary Material**

**Questionnaire handed to patients who received vaginal estrogen**

- Are you satisfied with the vaginal estrogen treatment?

Please give a note between 0 (not at all) and 10 (very well)

- Have you experienced any undesirable side effects with the current treatment (choose one or many of the following)
  - Irritability
  - Headaches
  - Insomnia
  - Nausea
  - Diarrhea
  - Redness/itching at the application site
  - Acne
  - Dry skin
  - Back pain
  - Mastalgia
  - Fatigue
  - Other (specify)
- Did you find there were one or many drawbacks for the current treatment? YES/NO
- Open-ended comments:

**Questionnaire handed to patients who received transdermal estrogen**

- Are you satisfied with the transdermal estrogen treatment?

Please give a note between 0 (not at all) and 10 (very well)

- Have you experienced any undesirable side effects with the current treatment (choose one or many of the following)
  - Irritability
  - Headaches
  - Insomnia
  - Nausea
  - Diarrhea
  - Redness/itching at the application site
  - Acne
  - Dry skin
  - Back pain
  - Mastalgia
  - Fatigue
  - Other (specify)
- Did you find there were one or many drawbacks for the current treatment? YES/NO
- Open-ended comments:
- Have you ever previously received vaginal estrogen treatment before an embryo transfer? YES/NO
- If yes, which route do you prefer? Vaginal or transdermal ?
- If you have already previously received vaginal estrogen before a frozen embryo transfer, how do the two treatments compare?
  - You experienced more undesirable side effects with transdermal estrogen compared to vaginal estrogen
  - You experienced less undesirable side effects with transdermal estrogen compared to vaginal estrogen
  - You experienced the same rate of undesirable side effects with transdermal and vaginal estrogen
  - You did not experience any undesirable side effects with transdermal and vaginal estrogen
